# A Rare Case of Cerebral Fat Embolism With No Respiratory or Dermatologic Involvement

**DOI:** 10.7759/cureus.22192

**Published:** 2022-02-14

**Authors:** Zakaria Salimi, Mehdi Ami Ali, Rim Tazi, Yasmine Mimouni, Asmaa Hazim, Jehanne Aasfara

**Affiliations:** 1 Neurology, Mohammed VI University of Health Sciences (UM6SS), Casablanca, MAR; 2 Emergency Medicine, Faculty of Medicine, Mohammed VI University of Health Sciences (UM6SS), Casablanca, MAR; 3 Neurology, Cheikh Khalifa Ibn Zayed Hospital, Mohammed VI University of Health Sciences (UM6SS), Casablanca, MAR; 4 Neurology, Cheikh Khalifa Bin Zayed Hospital, Casablanca, MAR; 5 Neurology, International Cheikh Khalifa University Hospital, Mohammed VI University of Health Sciences (UM6SS), Casablanca, MAR

**Keywords:** gurd's criteria, starfield pattern, mri, fat embolism syndrome, cerebral fat embolism

## Abstract

Fat embolism syndrome is potentially lethal. It is frequently a complication of long bone fractures and/or orthopedic surgery. Cerebral fat embolism is an unusual condition characterized by purely cerebral involvement. Neurological signs can be variable and brain MRI has a pivotal role in the diagnosis. We report the case of a 69-year-old male who presented motor impairment and a disorder of consciousness in the early postoperative course of total hip arthroplasty for a left femoral neck fracture, which occurred 24 hours before surgery. He had no dermatologic or respiratory signs. No respiratory or dermatologic signs were found. Blood samples showed moderate thrombopenia and hemolytic anemia. Multiple lesions were found on brain MRI. Diagnosis of cerebral fat embolism was established after ruling out differentials.

## Introduction

Fat embolism (FE) is a well-known complication of long bone fractures and orthopedic procedures [1]. Mechanical and biochemical pathophysiological theories are likely to be the underlying mechanisms [2]. The classic clinical triad of FE syndrome (FES) refers to pulmonary distress, neurologic symptoms, and petechial rash. Among literature, the incidence ranges from 1% (retrospective studies) to 19% (prospective studies) [3-6]. Cerebral FE (CFE) refers to the lodging of fat emboli within the brain’s microvasculature responsible for acute neurological signs. Although the real incidence of CFE is not well established due to the lack of prospective studies, it may be responsible for a high morbidity and mortality rate. MRI brain findings are the hallmark of CFE. As CFE can occur with no respiratory or dermatologic signs, early diagnosis remains crucial to initiate supportive strategies representing the mainstay of therapeutic management. We report a case of CFE with no respiratory or dermatologic signs after total hip arthroplasty for a left femoral neck fracture.

## Case presentation

A 69-year-old patient was admitted for motor impairment and altered consciousness, in the early postoperative course of total hip arthroplasty for a left femoral neck fracture that occurred 24 hours before surgery. The patient has a past medical history of type 2 diabetes mellitus, essential arterial hypertension, and ischemic heart disease.

On admission, the examination found a Glasgow Coma Scale of 13 (E4V4M5). The temperature was 38.1°C, the blood pressure was 130/80 mmHg, the heart rate was 85 beats per minute, the respiratory rate was 23 breaths per minute, and the oxygen saturation was 92% on pulse oximetry. Kernig and Brudzinski's signs were negative. Neurologic examination found right hemiparesis (motor power was at grade 4/5) with moderate expressive aphasia. NIHSS score of 13. There were no skin lesions. Laboratory blood tests on admission showed: Normochromic normocytic anemia (10.4 g/dL) with a possible hemolytic mechanism (high levels of bilirubin and LDH). Moderate thrombocytopenia (102.000/mm^3^). A hyperleukocytosis at 15.000/mm^3^. Erythrocyte sedimentation rate at 58. C-reactive protein (CRP) was 150 mg/L. He had normal ranges of hematocrit and no biological signs of kidney failure. Blood cultures were negative. We outline that no biological disorders were found on blood samples before this onset.

**Figure 1:**
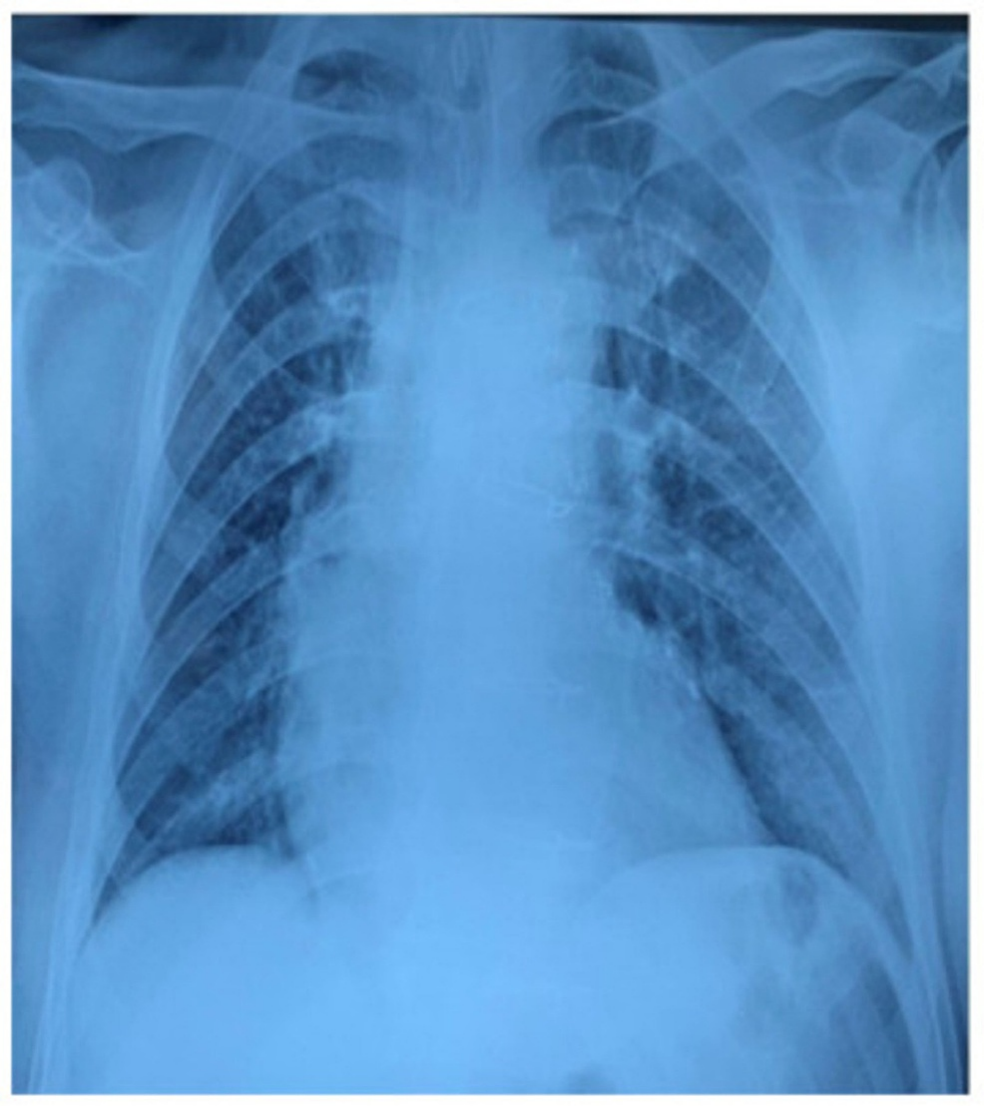
Chest x-ray showing clear lung fields.

Biological reassessment 48 hours later found serum creatinine levels reaching 21.1 mg/L (normal range 6-12 mg/L), and serum uremic level at 0.88 g/L (normal range 0.15-0.45 g/L). Arterial blood gases (ABGs) found Ph at 7.31 with normal rates of PaO_2_ and PaCO_2_. Brain computed tomography showed no abnormality. Chest x-ray showed clear field lungs (Figure 1). 

**Figure 2:**
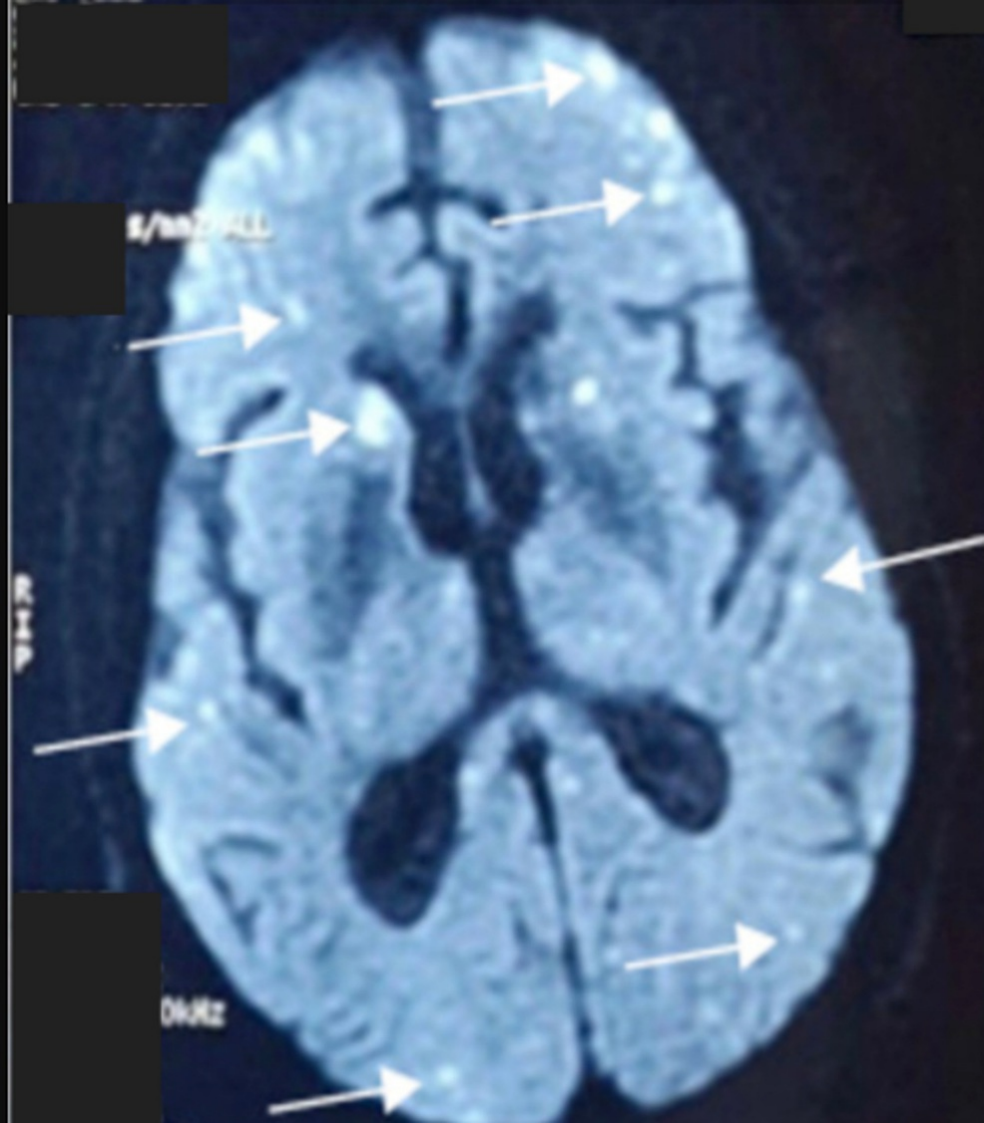
Brain MRI on diﬀusion-weighted (DWI) sequence: scattered hypersignals (“Starfield” pattern).

**Figure 3:**
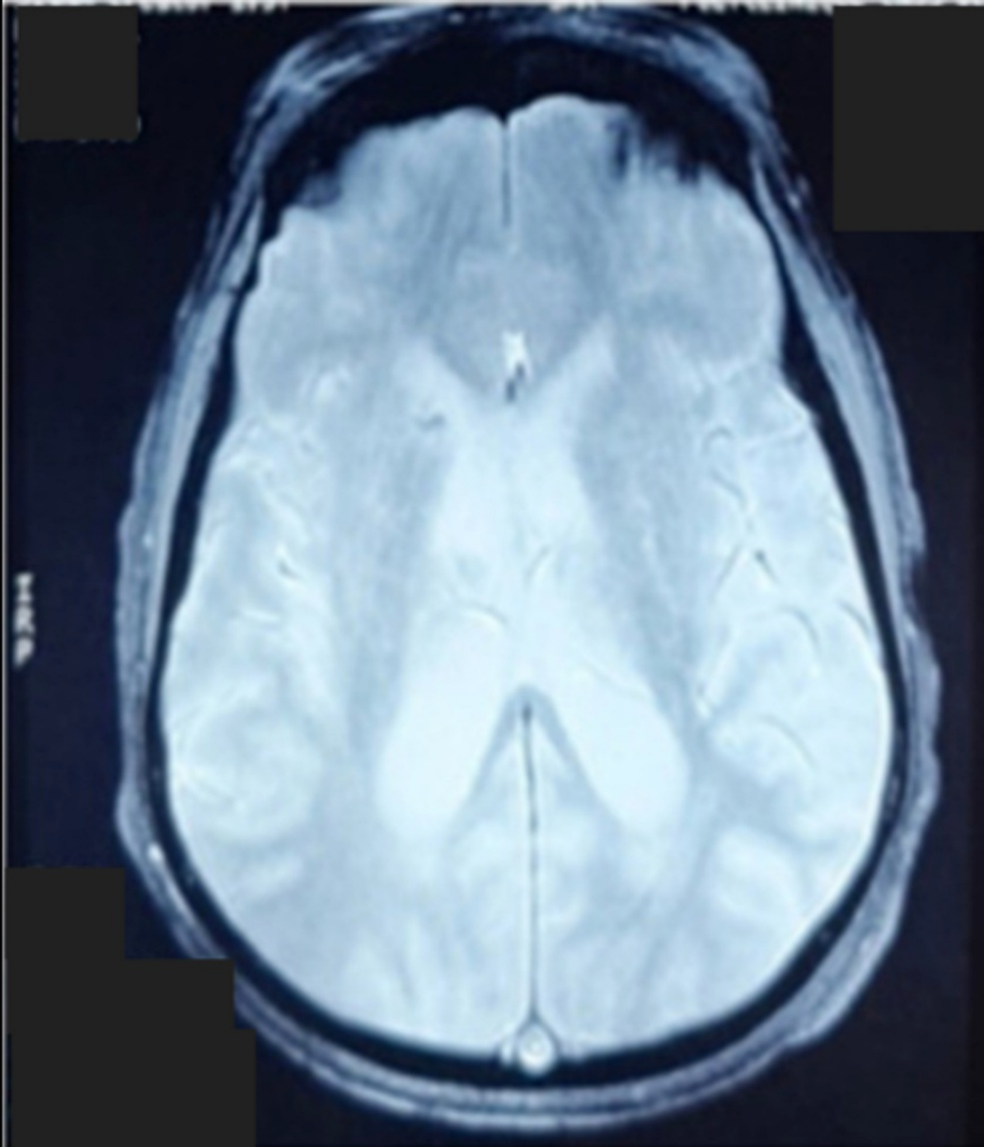
No petechial hemorrhage found on T2* sequences.

**Figure 4:**
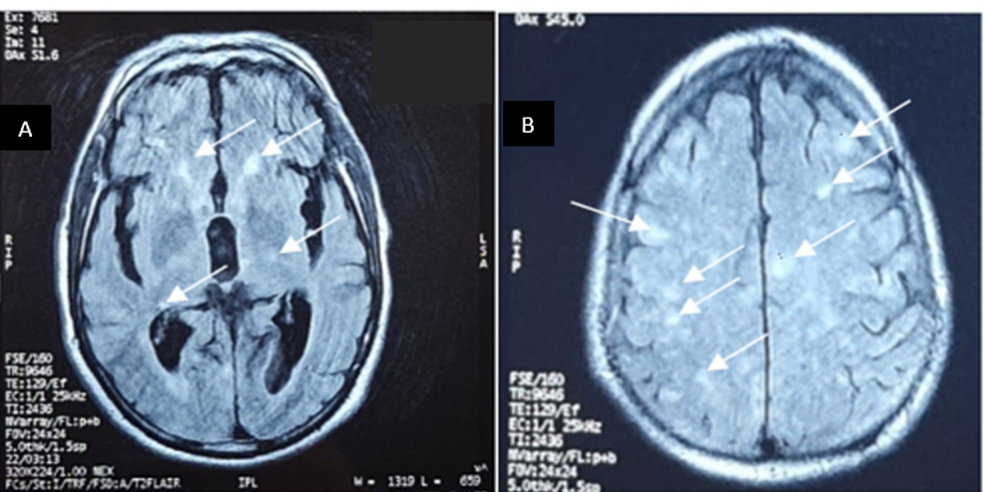
Diﬀuse hypersignal fluid-attenuated inversion recovery (FLAIR) lesions. (A) Periventricular areas. (B) Subcortical areas.

Electroencephalogram found no epileptic discharges. The cerebral MRI showed multiple lesions of restricted diffusion-weighted imaging (DWI) with a corresponding hypointense signal on apparent diffusion coefficient (ADC) with confluent white matter hyperintense lesions on the fluid-attenuated inversion recovery (FLAIR) sequence with no petechial hemorrhage found on T2* (Figures 2-4A, 4B).

 

 

Transthoracic echocardiogram (TTE) found a left ventricular ejection fraction of 40% (similar findings on TTE before the orthopedic procedure), with no thrombus, vegetations, or a patent foramen ovale. Transesophageal echocardiography (TEE) did not find other abnormalities. Ultrasonography of supra-aortic trunks revealed moderate intima-media thickness on carotids with no significant stenosis. A 24-hour ECG monitoring showed did not show any significant arrhythmia.

This patient’s clinical, laboratory, and imaging findings were compatible with a diagnosis of CFE with no respiratory signs. The patient had a good clinical recovery, regained full consciousness, and rapidly improved motor impairment. He showed no respiratory signs. Blood samples on the sixth day found normal ranges of biological parameters. The patient was discharged within 10 days.

## Discussion

The incidence of FE in autopsy studies ranges from 68% to 82% in blunt trauma patients [1,2]. FE usually collects in regions with rich microvasculature, such as the lungs, brain, and kidneys. Pathogenesis combines mechanical and biochemical theories. However, the exact mechanisms remain unknown [3].

As a clinical entity, FES occurs in 11% to 19% of patients with long bone fractures in prospective studies [4] and less than 1% in retrospective studies [5,6]. Christie et al. found echogenic material within cardiac cavities in 87% of tibia and femur fractures procedures after performing TEE in 111 orthopedic surgeries. This material was confirmed to be FE in only 33% of patients after blood sampling from the right atrium [7].

FES is referring to a clinical triad of acute respiratory failure, neurological disorders, and mucocutaneous petechial signs. This triad does not usually take place simultaneously. Symptoms frequently occur 12-72 hours after the initial trigger [4-6]. Hypoxemia is usually the first manifestation [8]. Biological results are nonspecific. Common blood disorders in FES are thrombocytopenia and hemolytic anemia [9].

Epidemiological findings support the theory that FES could be underrepresented. As symptoms can be variable and nonspecific in FES, there are no universal strict criteria, and diagnosis is usually based on exclusion. Tables 1-3 show the diagnostic criteria suggested by Gurd and Wilson [10], Schonfeld [11], and Lindeque [12]. In the current case, our patient did not fulfill the criteria.

**Table 1:** Gurd and Wilson’s criteria for FES diagnosis (two major criteria or one major criterion plus two minor criteria). FES - fat embolism syndrome

Major Criteria	Minor Criteria
Petechial rash	Tachycardia > 120 beats/min
Respiratory insufficiency	Fever
Cerebral involvement	Retinal changes: fat or petechiae
	Jaundice
	Renal signs: anuria or oliguria
	Thrombocytopenia
	Anemia
	High erythrocyte sedimentation rate
	Fat macroglobuliinemia

 

**Table 2:** Schonfeld’s criteria for FES diagnosis (total score of >5 is required for diagnosis). FES - fat embolism syndrome

Criteria	Points
Petechia	5
Chest x-ray change (diffuse alveolar change)	4
Hypoxemia (PaO_2_ < 9.3 kPa)	3
Fever (temperature > 38°C)	1
Tachycardia (HR > 120 bpm)	1
Tachypnea (>30/min)	1
Confusion	1

 

**Table 3:** Lindeque’s criteria for FES diagnosis. FES - fat embolism syndrome

Criteria
1. Sustained PaO_2_ < 8 kPa
2. Sustained PaCO_2_ > 7.3 kPa or pH < 7.3
3. Sustained respiratory rate > 35/min despite sedation
4. Increase work of breathing, dyspnea, accessory muscle use, tachycardia, and anxiety

CFE with no respiratory signs is rare but possible. Neurologic manifestations include cerebral ischemic or hemorrhagic stroke, seizures, autonomic dysfunction, and diffuse brain injury [8]. Neuroimaging is a crucial tool for the assessment of CFE. Five distinct brain MRI patterns have been reported through the acute, subacute, and late stages: 1) Scattered cytotoxic edema called “Starfield” pattern. 2) Confluent cytotoxic edema in white matter. 3) Vasogenic edema lesions that may enhance. 4) Petechial hemorrhage of white matter. 5) Chronic sequelae [2]. Locations usually involve the subcortical and periventricular white matter, corpus callosum, the basal ganglia, the brainstem, and the cerebellar [13].

Our patient developed CFE after a long bone fracture and total hip arthroplasty. He manifested neurological signs in the early postoperative course with no respiratory or cutaneous symptoms. Blood analysis found moderate thrombopenia and hemolytic anemia which represent usual biological findings in FES. Although the most sensitive observation was brain MRI findings consistent with “Starfield Pattern” (Hyperintense DWI dot-like lesions) and hyperintense FLAIR lesions consistent with vasogenic edema involving the white matter, caudate nucleus, and thalamic nucleus.

In 2012 Lee et al. suggested new modified Gurd’s criteria. These proposed criteria include brain MRI findings as more specific evidence of microembolic phenomenon [14], but have not yet been validated (Table 4) [15]. We underly that our patient fulfills the criteria. In our case, clinical, biological, and radiological observations have been linked to a possible CFE after ruling out differentials for multifocal cerebral emboli. Main differentials include cardiogenic and septic causes.

**Table 4:** Modified Gurd criteria for CFE diagnosis. Diagnosis of CFE requires brain MRI findings with one major + three minor or two major + two minor.

Major Criteria	Minor Criteria
Neurologic alteration	Tachycardia
Hypoxemia and/or bilateral pulmonary infiltrates	Fever
Petechia on the conjunctiva or upper trunk	Thrombocytopenia
	Anemia with coagulopathy or disseminated intravascular coagulation which is not explained by bleeding
	Renal involvement as oliguria or anuria
	Retinal infarct

Our case highlights the importance of considering CFE in patients presenting with isolated neurological signs in the early onset of trauma or orthopedic procedures. Brain MRI is a sensitive diagnostic tool with relevant findings. As the former criteria (Tables 1-3) do not include imaging findings, modified Gurd’s criteria may be beneficial for the early diagnosis of isolated CFE.

To our knowledge, there are no specific treatment guidelines either for CFE or for FES. Preventive and supportive measures represent the mainstay treatment of FES [15,16]. Early immobilization and stabilization may lower mortality and severe disability rates. Besides, venting the medullary canal during nailing may decrease the number of fat emboli [15]. In our case, surgery was carried out 24 hours after trauma. We believe that this delay may have increased the risk of CFE.

The use of intravenous dexamethasone is controversial. The beneficial effect could be linked to its anti-inflammatory role and capacity to reduce capillary permeability. Besides, adequate hemodynamic support, hypoxemia treatment, and septic state prevention are capital therapeutic goals. A dehydrating agent may be useful to protect brain function. Some authors recommend the use of sedative drugs to lower metabolism in the brain cells. The benefit of early hyperbaric oxygen therapy, increasing blood oxygen pressure, has been supported [16].

## Conclusions

Isolated CFE is a rare complication. Diagnosis should be considered in patients presenting with acute neurological manifestations in the early course of long-bone fractures or orthopedic procedures. Brain MRI should be performed to substantiate the clinical diagnosis. Modified Gurd’s criteria are more sensitive for early CFE diagnosis, which may lead to quickly initiating supportive measures. Further studies are required to improve the therapeutic management of FES and CFE.
